# The Mediational Role of Cognitive Emotion Regulation Strategies in the Relationship of Ego-strength and Adjustment to Infertility in Women

**Published:** 2015-06

**Authors:** Negar Teimourpour, Mohammad Ali Besharat, Abbas Rahiminezhad, Batool Hossein Rashidi, Masoud Gholamali Lavasani

**Affiliations:** 1 Department of Psychology, Faculty of Psychology and Educational Sciences, University of Tehran, Tehran, Iran; 2 Reproductive Health Research Centre, Tehran University of Medical Sciences, Tehran, Iran

**Keywords:** infertility, adjustment, ego-strength, emotion regulation

## Abstract

**Objective:** Infertility is considered as an intense and prolonged stressful experience. Despite of high prevalence of infertility and its emotional burden for couples and especially for women, the knowledge regarding psychological factors influencing adjustment to it is limited. The aim of the present study was to investigate the mediational role of cognitive emotion regulation strategies in the relationship of ego-strength and adjustment to infertility in women.

**Materials and methods:** A total number of 275 women with primary infertility referring to Valie-asr Reproductive Health Research Center (Tehran Imam Khomeini Hospital) participated in the present study. Data was collected via demographic information questionnaire, Ego-Strength Scale (ESS), Cognitive Emotion Regulation Questionnaire (CERQ) and Adjustment to Illness Scale (AIS). Data were analysed using Pearson correlation and path analysis methods Using SPSS (18) and LISREL (8.5) software.

**Results:** Results indicated there are significant positive correlation between ego-strength and adjustment to infertility (r = 0.44, p < 0.01). Also Adjustment has significant positive correlation withadaptive emotion regulation strategies (r = 0.38, p < 0.01) and significant negative correlation with non-adaptive emotion regulation strategies (r = -0.43, p < 0.01). Results of path analysis indicated emotion regulation strategies mediate the relationship of ego-strength and adjustment.

**Conclusion:** These results can be helpful in making preventive policies, identifying high risk patients and planning psychological interventions.

## Introduction

Infertility is defined as inability to conceive a child after one year of regular sexual encounter without using any contraception (1). It is estimated that one in every six couples experiences infertility (2). The inability to achieve pregnancy is highly stressful for couples and especially for women (3). Infertile women- especially those who receive infertility treatments- regularly report that preoccupation with infertility related issues are all consuming and burdensome which requires high levels of adjustment (4). Research has indicated that the level of stress in infertile women is equivalent to the stress of having Coronary Heart Disease (CHD), cancer and HIV positive (5). Infertile individuals experience various psychological problems. Many studies have found significant correlations between the experience of infertility and depression and anxiety (6), sexual dissatisfaction (7), general stress and infertility specific stress (8), decrease in well-being (9), decrease in quality of life (10), marital dissatisfaction (11), grief and loss (12), social withdrawal and isolation (13). 

Individuals, who receive infertility treatments, report higher levels of anxiety and emotional distress in comparison with public population, but this response is not general and some infertile individuals report more psychological distress in the experience of infertility (14). Psychological characteristics and variables can explain the difference in the reaction of women to infertility. One of the psychological constructs that can be helpful in explaining the way infertile women adjust to infertility is ego-strength.Ego-strength refers to the ability of successfully managing instinctual needs, internal inhibitions and social reality (15). Block and Block (16) introduced two main sub-scales of ego-strength: ego-control and ego-resiliency. Ego-control refers to the ability of altering one’s own responses in line with the standards, ideals, values and social expectations and supporting them in order to achieve long term goals. Ego-resiliency refers to the way person responses to the internal and external stressful events. Individuals with high ego-resiliency, adapt quickly and have the ability of planning based on long-term goals, recover faster from traumas and experience less anxiety and distress in the face of stressful events. Ego-control and ego-resiliency both affect ego-strength and are indexes of maturation which lead to the ability of faster adjustment with the internal and external world (16).

Emotion regulation is another variable that can be helpful in explaining the way women adjust to infertility. Emotion regulation refers to managing the behavior, cognition, attention, and physiological processes with the purpose of terminating, altering, expressing or maintaining the emotional experience (17). Cognitive emotion regulation strategiesare a strong and specific category in the area of emotion regulation (18) and refer to the cognitive strategies and processes used to manage the emotions (19). Research has shown that psychological distress experienced in stressful life events have significant correlations with cognitive emotion regulation strategies and these strategies have a significant role in the development of emotional and behavioural problems after facing stressful events (18). Emotion regulation also has an important role in the process of adjustment to infertility. Infertility is an experience that leads to decrease in the function of regulation of emotions and causes in increase of negative affect in women (20).

Ego-strength can affect emotion regulation and eventually adjustment. The ability of emotion regulation is an index of ego-strength and an appropriate level of function of ego (21). Research has shown individuals with higher levels of ego-resiliency use more adaptive emotion regulation strategies facing stressful life events and tasks (22). Higher levels of ego-control lead to increase in the ability of regulating emotions and decrease in negative emotionsin individuals. On the other hand, lower levels of ego-control decreases the ability of activating of behaviours that regulate the emotions (23).

Regarding the stated background for the important role of psychological factors in adjustment to infertility in women, the aim of the present study is to assess the relationship between adjustment to infertility with ego-strength and cognitive emotion regulation. Also the mediational role of emotion regulation in the relationship of ego-strength and adjustment to infertility in women is investigated.

## Materials and methods

A total number of 275 women with primary infertility referring to Valie-asr Reproductive Health Research Center (Tehran Imam Khomeini Hospital) participated in the present study according to the available sampling method. Including criteria were: being female, primary infertility diagnosis, complete consent to participate in the research and being literate. Excluding variable were: diagnosis of a major debilitating internal or surgical illness, major psychiatric disorder and consumption of psychoactive drugs. Women participated in the research with complete consent and before participating in the study, a brief explanation of the purpose of the study was explained to them. Questioners were presented personally and it was explained that the results of the research is confidential and will be used only for the scientific purposes. The researcher presented and received the questionnaires and answered the questions of the participants. The questionnaires were to be filled out within 10-15minutes. Pearson correlation and path analysis methodswere used to analyse the data. Data was analysed using SPSS (18) and LISREL (8.5) software. The questionnaires which were used to collect the date were:

     1. Demographic and personal information questionnaire: This questionnaire was designed by the researchers and assessed demographic variables, including and excluding variables.

     2. Ego-strength Scale (ESS): Ego-strength Scale has 25 items and assesses the level of the strength of the ego in managing difficult life situations in a likert scale (1 to 5) and has been normalized in Iranian population (24). Minimum and maximum scores of the total score of the scale for each individual are 25 and 125. Psychometrics properties of Ego-Strength Scale has been assessed and confirmed in Iranian population in various studies in both clinical (n = 372) and normal (n = 1257) groups (24). In these studies, a Cronbach alpha coefficient was from 0.81 to 0/93 for the total score of the scale. This result confirmed internal consistency of the total score of the scale. Test-retest reliability was assessed in clinical sample (n = 122) and normal group (n = 274) with 2 to 4 weeks intervals. Results indicated correlations from 0.73 to 0.88 for the total score of the scale. This result is significant (p < 0.001) and confirms test-retest reliability of Ego-Strength Scale (24).

     3. Cognitive Emotion Regulation Questionnaire (CERQ): Cognitive Emotion Regulation Questionnaire has 18 items and assesses adaptive and non-adaptive cognitive emotion regulation strategies in response to threatening and stressful life events and experiences in a likert scale (0 to 5) with nine sub-scales: self-blame, other-blame, focus on thought/rumination, catastrophizing, putting into perspective, positive refocusing, positive reappraisal, acceptance and refocus on planning. Minimum and Maximum scores of each sub-scales are 0 and 2. Psychometric properties of the scale have been confirmed in various studies (18). In preliminary investigation of psychometric properties of the scale in a sample of Iranian population (n = 368), Cronbach alpha was from 0.67 to 0.89. These results confirm the internal consistency of the scale. Test-retest reliability in a number of the participants of the study (43 female and 36 male) with 4 weeks interval was from r = 0.57 to r = 0.76. These correlations were significant (p < 0.001) and confirm test-retest reliability of the scale. Content validity of the scale was assessedby using correlation between scores of 15 psychology experts. Kendal coefficient for sub-scales was from 0.81 to 0.92 (p < 0.001) (25).

     4. Adjustment to Illness Scale (AIS): Adjustment to Illness Scale has 12 items and assesses adjustment to medical conditions in a likert scale (0 to 6). Minimum and maximum scores in this scale are 0 and 72. Psychometric properties of the scale has been assessed and confirmed in various illnesses (Coronary-Heart disease, Multiple Sclerosis, chronic pain, and infertility) in Iranian population. Cronbach Alpha scores of the scalein different studies has been assessed and confirmed and is between 0.71 and 0.87. Convergent and discriminant validity of adjustment to illness scale has been assessed and confirmed in various studies by concurrent administration with Mental Health Inventory (MHI), Hospital Anxiety and Depression Scale (HADS) and Positive Affect Negative Affect Scale (PANAS). There were significant (p < 0.001) correlations between adjustment and psychological well-being (r = 0.65), positive affect (r = 0.59), psychological distress (r = -0.51) and negative affect (r = -0.57). Predictive validity of adjustment to illness scale was assessed by comparison of a normal and patient group. Results indicated scores of adjustment are sensitive (p < 0.001) in discriminating two groups. Results of exploratory and confirmatory factor analysis, confirmed construct validity by determining one overall factor (26).

## Results

The mean of age of the participants was 31.8 years± 6.6 (standard deviation), mean of marital duration was 87 months ± 60.2 (standard deviation) and mean of infertility duration was 47.2 months ± 40 (standard deviation). Descriptive characteristics (mean and standard deviation) and correlational matrix among variables of the study are presented in table 1.

Results of the Pearson correlation analysis indicate there are significant correlations between ego-strength and adjustment to infertility (r = 0.44, p < 0.01), adaptive emotion regulation strategies and adjustment to infertility (r = 0.38, p < 0.01), non-adaptive emotion regulation strategies and adjustment to infertility (r = -0.43, p < 0.01).

Results of the path analysis are demonstrated in the figure 1.Standard solutions are shown in this diagram and they are all significant (p < 0.05).

Fit indices of the model were: RMSEA = 0.02, x^2^/df = 1.23, GFI = 1, AGFI = 1, CFI = 1, NFI = 1. These results indicate that the model perfectly fits the data.

**Table 1:** descriptive characteristics and correlational matrix among variables of the study

	**1**	**2**	**3**	**4**
ego-strength	1			
Adaptive emotion regulation	0.54**	1		
Non-adaptive emotion regulation	-0.53**	-0.33**	1	
Adjustment	0.44**	0.38**	-0.43**	1
Mean	78.3	3.1	2.9	32.7
Standard Deviation	14.7	0.7	0.77	11.4

** p < 0.01

**0.19Figure 1:**
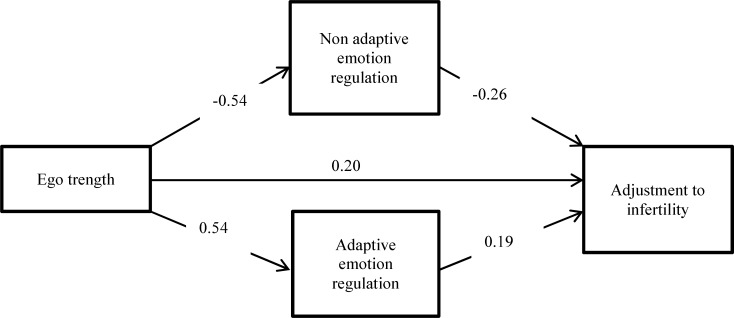
Results of the path analysis of the mediational role of cognitive emotion regulation in the relationship of ego-strength and adjustment to infertility based on standard solution

**Table 2:** Direct, indirect and total effects of ego-strength on adjustment with the mediation of emotion regulation strategies

**Path**	**Direct**	**Indirect**	**Total**
Ego-strength	0.16 (2.87)	0.19 (4.91)	0.34 (8.7)
	p < 0.05	p < 0.05	p < 0.05

Direct, indirect and total effects of ego-strength on adjustment are presented in table 2. These results are based on β coefficients. T values are shown in parentheses in front of the β coefficients and p values are shown under them.

Direct, indirect and total effect of ego strength on adjustment are all significant (p < 0.05). The indirect effect of ego-strength on adjustment (0.19) is biggerthan the direct effect (0.16). This result indicates that adaptive and non-adaptive cognitive emotion regulation strategies mediate the relationship between ego-strength and adjustment to infertility in women

## Discussion

The results of the present study indicated ego-strength has a significant positive correlation with adjustment to infertility in women. Previous research has shown that individuals with low ego-strength are more prone to psychopathology, in comparison with individuals with higher levels of ego-strength (15). Research has shown that ego-strength has an important role in predicting health and represents the individual’s attitude about the problem, reaction to frustration and the ability of adaptation with one’s emotional profile. Ego-strength has an important role in predicting health and treatment adherence in chronic conditions (27). Individuals with higher levels of ego-strength, experience more positive effects, higher levels of self-confidence and higher psychological adjustment in comparison with individuals with lower levels of ego-strength (28). Individuals with higher levels of ego-strength don’t experience distress and emotional difficulties in the face of stressful and difficult tasks of life and that is because of their flexible approach to life (22). 

The results of the present study indicated infertile women who use adaptive cognitive emotion regulation strategies have higher levels of adjustment to infertility and women who use non-adaptive cognitive emotion regulation strategies have lower levels of adjustment to infertility. Previous research has shown that deficits in emotion regulation have a key role in development of psychopathology, especially internalizing problems, such as depression and anxiety (29). Repeated and long term use of non-adaptive cognitive emotion regulation strategies leads to exacerbation of stress, and eventually depression and anxiety. On the other hand, using adaptive emotion regulation strategies leads to decrease in negative affect and better adjustment to problems (30). The experience of infertility causes decrease in positive affect and increase in negative affect and emotion regulation deficiencies in women (20). 

The result of the present study indicated that adaptive and non-adaptive cognitive emotion regulation strategies mediate the relationship of ego-strength and adjustment to infertility in women. Previous research has shown ego-strength predicts emotion regulation (22, 23) and ability of adaptively regulation emotions is an index of strength of ego-strength (21). Individuals with higher levels of ego-resiliency use coping strategies which increases positive emotion and this leads to decrease in the stress. These individuals are more skilled in implementing positive emotions and because they repeatedly experience positive emotions, they have better and easier access to them. Repeated and easy access to positive emotions requires minimum levels of cognitive resources and energy for the activation of them, therefore activation of positive emotions-even in highly stressful situations- is very easy and effortless for individuals with high levels of ego-resiliency. In conclusion, higher levels of ego-strength lead to using more adaptive emotion regulation strategies and eventually higher levels of adjustment in individuals (31).

The results of the present study highlight the significant relationship between ego-strength andcognitive emotion regulation strategies with adjustment to infertility. The path analysis results indicated, the indirect effect of ego-strength on adjustment (with the mediation of emotion regulation) is bigger than the direct effect and based on these findings we can conclude that ego-strength affects adjustment to infertility in women indirectly, with the mediation of adaptive and non-adaptive emotion regulation strategies. These results can be helpful in making preventive policies, identifying high risk patients and planning psychological interventions.

The results of the present study should be interpreted with considering the limitations of the study. First, self-report measures were used to gather the data. Second, only women participated in the study. As a result, it is suggested to use qualitative methods and also include men and couples in the future studies.

## References

[1] Zegers-Hochschild F, Adamson GD, de Mouzon J, Ishihara O, Mansour R, Nygren K (2009). International Committee for Monitoring Assisted Reproductive Technology (ICMART) and the World Health Organization (WHO) revised glossary of ART terminology, 2009. Fertil Steril.

[2] World Health Organization (2010). Mother or nothing: the agony of infertility. Bull World Health Organ.

[3] Cousineau TM, Domar AD (2007). Psychological impact of infertility. Best Pract Res Clin Obstet Gynaecol.

[4] Redshaw M, Hockley C, Davidson LL (2007). A qualitative study of the experience of treatment for infertility among women who successfully became pregnant. Hum Reprod.

[5] Domar AD, Clapp D, Slawsby EA, Dusek J, Kessel B, Freizinger M (2000). Impact of group psychological interventions on pregnancy rates in infertile women. Fertil Steril.

[6] Ogawa M, Takamatsu K, Horiguchi F (2011). Evaluation of factors associated with the anxiety and depression of female infertility patients. Biopsychosoc Med.

[7] Tao P, Coates R, Maycock B (2011). The impact of infertility on sexuality: A literature review. Australas Med J.

[8] Van den Broeck U, D'Hooghe T, Enzlin P, Demyttenaere K (2010). Predictors of psychological distress in patients starting IVF treatment: infertility-specific versus general psychological characteristics. Hum Reprod.

[9] Bennett SLR (2009). An investigation of sources of women’s infertility-specific distress and well- being [Thesis of doctor of philosophy].

[10] Lau JT, Wang Q, Cheng Y, Kim JH, Yang X, Tsui HY (2008). Infertility-related perceptions and responses and their associations with quality of life among rural Chinese infertile couples. J Sex Marital Ther.

[11] Daniluk JC, Trench E (2007). Long - term adjustment of infertile couples following unsuccessful medical interventions. J counseling development.

[12] Peterson BD, Eifert GH (2011). Using Acceptance and Commitment Therapy to treat infertility stress. Cognitive Behavioral Pract.

[13] Watkins KJ, Baldo TD (2004). The infertility experience: Biopsychosocial effects and suggestions for counselors. J Counseling Development.

[14] Rashidi B, Montazeri A, Ramezanzadeh F, Shariat M, Abedinia N, Ashrafi M (2008). Health-related quality of life in infertile couples receiving IVF or ICSI treatment. BMC Health Services Research.

[15] Mccrae RR, Costa PT (2005). Personality in adulthood.

[16] Block JH, Block J, Collins WA (1980). The role of ego-control and ego-resiliency in the organization of behavior.

[17] Gross JJ (2010). Emotion regulation in adulthood: timing in everything. Curr Dir Psych Sci.

[18] Garnefski N, Kraaij V (2006). Cognitive emotion regulation questionnaire: Development of a short 18-item version (CERQ-short). Pers Individ Dif.

[19] Garnefski N, Kraaij V, Spinhoven P (2001). Negative life events, cognitive emotion regulation and emotional problems. Pers Indiv Differ.

[20] Dana S, Narimani M, Mikaeili N (2013). Comparison of emotion regulation and emotion control in fertile and infertile women. Intl J Phys Beh Res.

[21] Pellitteri J (2009). Emotional Processes in Music Therapy.

[22] Caldwell JG, Shaver PR (2012). Exploring the cognitive-emotional pathways between adult attachment and ego-Resiliency. Indiv Diff Res.

[23] Abela JRZ, Hankin BL (2008). Handbook of depression in children and adolescents.

[24] Besharat MA (2006). Development and validation of Ego-Strength Scale. Research report.

[25] Besharat MA, Bazzazian S (2014). Psychometric properties of Cognitive Emotion Regulation Strategies Questionnaire in a sample of Iranian population. Journal of Shahid Beheshti School of Nursing & Midwifery.

[26] Besharat MA (2000). Development and validation of Adjustment to Illness Scale. Research report.

[27] Settineri S, Mento C, Santoro D, Mallamace A, Bellinghieri G, Savica V (2012). Ego strength and health: An empiric study in hemodialysis patients. Health.

[28] Letzring TD, Block J, Funder D (2005). Ego-control and ego-resiliency: Generalization of self-report scales based on personality descriptions from acquaintances, clinicians, and the self. Journal of Research in Personality.

[29] Asberg K (2013). Hostility/anger as a mediator between college students’ emotion regulation abilities and symptoms of depression, social anxiety, and generalized anxiety. J Psychol.

[30] D’Avanzato K, Joormann J, Siemer M, Gotlib IH (2013). Emotion regulation in depression and anxiety: examining diagnostic specificity and stability of strategy use. Cognitive Ther Res.

[31] Tugade MM, Fredrickson BL (2007). Regulation of positive emotions: Emotion regulation strategies that promote resilience. J Happiness studies.

